# Role of serum- and glucocorticoid-inducible kinase-1 in regulating torsion-induced apoptosis in rats

**DOI:** 10.1111/j.1365-2605.2010.01091.x

**Published:** 2011-08

**Authors:** Y-M Cho, H-F Pu, W J Huang, L-T Ho, S-W Wang, P S Wang

**Affiliations:** * Department of Physiology, School of Medicine, National Yang-Ming UniversityTaipei; † Department of Urology, School of Medicine, National Yang-Ming UniversityTaipei; ‡ Division of Urology, Department of Surgery, Taipei Veterans General HospitalTaipei; § Department of Medical Research and Education, Taipei Veterans General HospitalTaipei; ¶ Department of Physiology and Pharmacology, Chang-Gung UniversityTaoyuan, Taiwan

**Keywords:** apoptosis, FOXO3a, phosphoinositide-dependent protein kinase-1, serum- and glucocorticoid-inducible kinase-1, testicular torsion, testosterone

## Abstract

Serum- and glucocorticoid-inducible kinase-1 (SGK1) is a serine/threonine protein kinase that responds to various stimuli and mediates cell survival. Although it is known that testicular torsion leads to testicular damage and male infertility, the role of SGK1 in torsion remains unclear. This study investigated whether torsion-induced apoptosis is associated with changes in phosphoinositide-dependent protein kinase-1 (PDK1), SGK1 and forkhead transcription factor FOXO3a expression and/or phosphorylation in rats. Sprague-Dawley rats were divided into four groups: sham (control), 1, 2 and 4 h of unilateral torsion. Bilateral testes, testicular interstitial fluid (TIF) and blood samples were collected immediately after torsion. Our results revealed that SGK1 protein and mRNA were abundantly present in testes and were induced by 2 h of torsion, but that phosphorylation of SGK1, PDK1 and FOXO3a decreased simultaneously. After 2 h of torsion, the testosterone secretion capacity of the primary Leydig cells and testicular interstitial cells (TICs) was impaired and apoptotic spermatogonia and TICs were observed; in addition, the mean seminiferous tubular diameter was decreased. Torsion increased plasma corticosterone levels, but decreased plasma luteinizing hormone and testosterone levels. However, the testosterone levels of the TIF in the ipsilateral testes were significantly enhanced after 2 h of torsion, but suppressed in the contralateral testes. This animal study suggests that PDK1, SGK1 and FOXO3a are involved in torsion-induced apoptosis and that medical therapy should be performed as early as 2 h after the occurrence of torsion to prevent further damage.

## Introduction

Testicular torsion leads to infertility (Filho *et al.*, 2004); it is caused by spermatic cord twisting (Turner & Brown, 1993; Turner *et al.*, 2005). This obstruction of blood supply to the testes creates a condition in which there is a shortage of oxygen and nutrients from the blood and this results in testicular cell damage. Previous evidence from various ischaemia/reperfusion models has indicated that both necrosis and apoptosis are mechanisms for ischaemia-/reperfusion-induced cell death (Shiraishi *et al.*, 2000, 2001). Specifically, necrosis rarely happens to testes examined immediately after torsion, but it appears late after prolonged ischaemia and ischaemia/reperfusion. A previous study indicated that testicular ischaemic injury produced by 1080° torsion did not lead to significant macroscopic or histological testicular necrosis after 1–4 h of torsion. Moreover, the testicular necrosis after 60 days detorsion was similar between groups with different periods of torsion (Romero *et al.*, 2009). However, the detailed apoptotic mechanisms induced by up to 4 h testicular torsion remain to be determined.

The expression of serum- and glucocorticoid-inducible kinase-1 (SGK1), a serine threonine kinase, is mediated by a variety of extracellular stimuli (Richards, 2001); it controls cell volume, proliferation and apoptosis (Firestone *et al.*, 2003; Lang *et al.*, 2006). In cardiomyocytes, the inhibition of SGK1 activity can increase apoptosis from hypoxia or serum deprivation in vitro (Aoyama *et al.*, 2005). SGK1 phosphorylation and activation exerts its anti-apoptotic activity through the inactivation of pro-apoptotic proteins such as forkhead transcription factor FOXO3a (also known as FKHRL1). FOXO3a has been shown to be negatively regulated by SGK1, the effect of which is to limit the arrest and apoptosis in the FOXO3a-dependent cell cycle (Brunet *et al.*, 2001). Previous reports have shown that gene silencing with FOXO3a siRNA reverses oxygen-glucose deprivation-induced endothelial cell apoptosis and increases survival (Chong & Maiese, 2007), but that enhancing the phosphorylation of FOXO3a protects the mouse brain from ischaemic injury (Shioda *et al.*, 2007). Moreover, to activate the SGK-mediated cell survival cascade, SGK1 binds to 3-phosphoinositide-dependent protein kinase-1 (PDK1) and induces phosphorylation at Thr^256^ by PDK1 (Kobayashi & Cohen, 1999; Park *et al.*, 1999). This PDK1-dependent phosphorylation at SGK1 Thr^256^ is essential for the maximum stimulation of SGK1 activity (Biondi *et al.*, 2001). However, the effects of ischaemia on the expression of PDK1, SGK1 and FOXO3a in vivo have not been studied. Hence, it would be a great interest to explore PDK1/SGK1/FOXO3a signal cascade in ischaemia-induced apoptosis.

Testicular torsion is a typical ischaemic model that directly leads to male infertility (Filho *et al.*, 2004). The direct effects of testicular ischaemia on PDK1, SGK1 and FOXO3a have remained uninvestigated. Thus, we performed this animal study with the hypothesis that the PDK1/SGK1/FOXO3a signalling pathway is associated with testicular ischaemia-induced apoptosis in rats.

## Materials and methods

### Animals

Eight-week-old Sprague–Dawley male rats were maintained under controlled temperature (22 ± 2 °C) and light (06:00–20:00 hours) conditions, with food and water ad libitum. The use of the animals was approved by the Institutional Animal Care and Use Committee of National Yang-Ming University. All animals received human care in compliance with the *Principles of Laboratory Animal Care* and the *Guide for Care and Use of Laboratory Animals* (National Science Council, Taiwan).

### Testicular torsion

Seventy-two male rats were divided randomly into four groups: sham (control group, *n* = 16), 1 h (*n* = 16), 2 h (*n* = 20) and 4 h (*n* = 20) of unilateral torsion. A left longitudinal scrotal incision was made under an intraperitoneal injection of pentobarbital (50 mg/mL/kg) to expose the left testis as described elsewhere (Yang *et al.*, 2007). The left testis was twisted by turning it 720° counterclockwise, whereupon it was suture-fixed to the scrotal wall, to maintain the torsion state. Immediately after 1, 2 or 4 h of torsion, both the ipsilateral testis with torsion and the contralateral testis without torsion were removed and harvested for various measurements.

### The functional capacity of the TICs and LCs to secrete testosterone

The preparation of testicular interstitial cells (TICs) and Leydig cells (LCs) has been described elsewhere (Huang *et al.*, 2001). The preparations of TICs and LCs were found to contain approximately 20% and 85% Leydig cells (Lin *et al.*, 1998), respectively. TICs (1 × 10^6^ cells) and LCs (1 × 10^5^ cells) were seeded into tubes, then challenged with human chorionic gonadotropin (hCG) (0 and 0.05 IU/mL) at 34 °C for 1 h. After being centrifuged at 50 ***g*** for 10 min, the supernatant was collected for testosterone radioimmunoassay (RIA).

### Blood sampling and TIF collection

Blood samples were collected by heart puncture immediately after removal of the testes. The bilateral testes had their tunica albuginea pierced at the distal pole several times; then, the testicular interstitial fluid (TIF) was collected by centrifuging at 50 ***g*** for 15 min at 4 °C (Turner *et al.*, 1984).

### Histological examination

To determine morphological changes, the testes were fixed in Bouin's solution, dehydrated in an ethanol series and embedded in paraffin immediately after torsion. A number of 3-μm testes sections were prepared and then stained with haematoxylin and eosin (H&E). To measure the mean seminiferous tubular diameter (MSTD), each testis was assessed by measuring at least 25 separate tubular diameters, selecting the smallest and roundest seminiferous tubules in the field (Cosentino *et al.*, 1986).

### Measurement of apoptosis

Paraffin sections were prepared and then analysed using the DeadEnd Fluorometric TUNEL (terminal deoxynucleotidyl transferase-mediated dUTP-biotin nick-end labelling) System (Promega, Madison, WI, USA). Immunofluorescence was observed using a Leica TCS SP2 confocal microscope (Leica Microsystems, Heidelberg, Germany). The average percentage of apoptotic tubules was estimated by examining 100 cross-sections of seminiferous tubules from each specimen. The seminiferous tubules that contained at least one TUNEL-stained nucleus were considered apoptotic (Yazawa *et al.*, 2001).

### Testosterone RIA

The concentrations of plasma and TIF testosterone were measured by RIA as described elsewhere (Wang *et al.*, 1999; Chiao *et al.*, 2002). The sensitivity of the testosterone RIA was 2 pg per tube. The intra- and inter-assay coefficients of variation were 4.1% (*n* = 6) and 4.7% (*n* = 10), respectively.

### Corticosterone RIA

The concentration of plasma corticosterone was determined by RIA as described elsewhere (Pu *et al.*, 2006; Chang *et al.*, 2008). The sensitivity of corticosterone detection was 5 pg/mL. The intra- and inter-assay coefficients of variation were 3.4% (*n* = 5) and 9.5% (*n* = 5), respectively.

### Determination of plasma LH levels

Plasma luteinizing hormone (LH) levels were analysed according to the manufacturer's protocol using the rat pituitary kit MILLIPLEX MAP by the Luminex xMAP technology (Millipore Corp., St Charles, MO, USA). The sensitivity of the bioassay was 4.9 pg/mL for LH. The intra- and inter-assay coefficients of variation were 15% (*n* = 12) and 9.5% (*n* = 5), respectively.

### Immunoblotting

Testicular tissue suspensions were extracted with lysis buffer and disrupted by sonication. The protein extracts (20 μg) were separated by 8% sodium dodecyl sulphate-polyacrylamide gel electrophoresis and transferred to polyvinylidene fluoride (PVDF) membrane. The following antibodies were used for immunoblotting: p-SGK1 Thr^256^, FOXO3a and SGK1 (Upstate Biotechnology, Lake Placid, NY, USA); p-SGK1 Ser^422^ (Santa Cruz Biotechnology, Santa Cruz, CA, USA); p-PDK1 Ser^241^, PDK1 and p-FOXO1 Thr^24^/FOXO3a Thr^32^ (Cell Signaling, Danvers, MA, USA); and β-actin (Chemicon, Temecula, CA, USA). An enhanced chemiluminescence kit (Amersham, Piscataway, NJ, USA) was used for immunodetection. The protein bands were quantified using the Image J software (NIH, Bethesda, MD, USA).

### RNA extraction, RT-PCR and real-time PCR

Total RNA was extracted with Trlzol (Invitrogen, Carlsbad, CA, USA; Hsia *et al.*, 2006). Reverse transcription (RT) and polymerase chain reactions (PCR) used SuperScript III Reverse Transcriptase (Invitrogen) and Taq DNA polymerase master mix (Ampliqon, Copenhagen, Denmark), respectively. The applied RT-PCR primers, the expected PCR product lengths and the thermal conditions are described elsewhere (Tsai *et al.*, 2002). The hypoxanthine phosphoribosyltransferase gene (*HPRT*) was used as an internal control. The PCR products were size-fractionated on 2% agarose gels, and then stained with ethidium bromide before being photographed. The signals were measured using Image J software.

Real-time PCR was performed using the ABI PRISM 7700 sequence detection system with the TaqMan method (Applied Biosystems, Foster City, CA, USA). The *sgk1* and *HPRT* primers and the fluorogenic probe (Hsu *et al.*, 2009) were designed by Primer Express software and purchased from Applied Biosystems. The thermal conditions were 2 min at 50 °C and 10 min at 95 °C, followed by 40 cycles at 95 °C for 15 s and 60 °C for 1 min. The relative quantity of mRNA was estimated using a standard curve that was created by serial dilution of the RT product from control samples. The quantitative analysis of *sgk1* gene expression was normalized to that of the *HPRT* gene expression.

### Immunofluorescence microscopy

Testicular paraffin sections were prepared after torsion. A DNA marker and antibodies were used for immunostaining: 4,6-diamidino-2-phenylindole (DAPI), Rhodamine Red-X goat anti-rabbit immunoglobulin G (Jackson ImmunoResearch laboratories, Inc., West Grove, PA, USA), SGK1, FOXO3a and p-FOXO3a Thr^32^ (Abcam plc, Cambridge, UK). The sections were mounted using Vectashield mounting medium (Vector Laboratories, Burlingame, CA, USA). Immunofluorescence was observed using a Leica TCS SP2 confocal microscope (Leica Microsystems, Heidelberg, Germany).

### Statistical analysis

Each result is represented as the mean ± SEM of at least three independent experiments. Data were processed by one-way analysis of variance (anova). Multiple comparisons were performed by Student–Newman–Keuls test whenever one-way anova was significant (*p*<0.05). SigmaStat (Systat Software Inc., Chicago, IL, USA) was used for data analysis.

## Results

### Torsion-induced pathological changes

**Figure 1:**
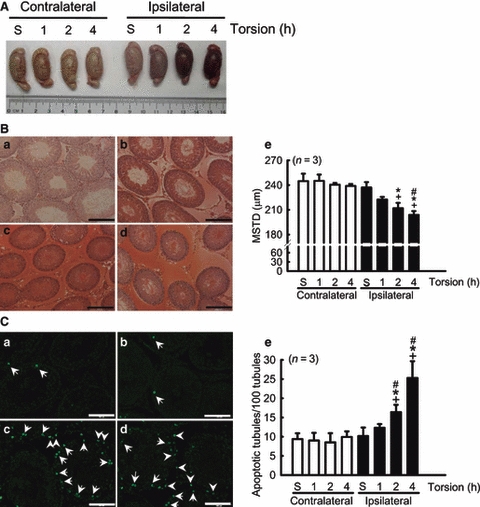
Apparent histological changes and apoptotic cells. (A) After 1 h of torsion, the ipsilateral testis showed congestion and haemorrhage and this was aggravated after 2 and 4 h of testicular ischaemia. (B) Oedema and haemorrhage were observed in the interstitial spaces, after (c) 2 h and (d) 4 h of torsion. Histological sections of the rats from the (a) sham and (b) 1-h groups show normal seminiferous tubular architecture (stain: haematoxylin and eosin; scale bar, 200 μm). (e) The quantifications of mean seminiferous tubular diameter of bilateral testes. (C) Testis sections stained by the terminal deoxynucleotidyl transferase-mediated dUTP-biotin nick-end labelling (TUNEL) method demonstrate the presence of apoptotic cells in testes (a–d). Prominent apoptotic spermatogonia (a–d, arrows) and interstitial cells (c and d, arrow head) in the ipsilateral testes are indicated. (e) Quantitative comparisons of TUNEL-stained cells after different periods of torsion (scale bar, 100 μm). Values are mean ± SEM. *p<0.05, vs. respectively contralateral testes; ^+^*p*<0.05, vs. sham (S) group; ^#^*p*<0.05, vs*.* ipsilateral 1 h group.

The ipsilateral testes showed congestion and haemorrhage after 1 h of torsion, and this became more obvious after 2 and 4 h of torsion (Fig. 1A). Microscopic images of the architecture of ipsilateral testicular seminiferous tubules are shown in Fig. 1B: a–d. The interstitial spaces displayed oedema after 2 and 4 h of torsion. In addition, the MSTD of the ipsilateral testes at 1, 2 and 4 h of torsion were 5, 13.5 and 22.5% lower than that of the sham group, respectively. Compared with the contralateral testis, 2 and 4 h of torsion significantly reduced the MSTD of the ipsilateral testis. The MSTD of ipsilateral testis at 4 h was also less than that of the 1-h group (Fig. 1B: e).

### Torsion-induced apoptosis

The percentage of apoptotic seminiferous tubules in the testes was detected by TUNEL assay (Fig. 1C: a–d) followed by a quantitative comparison (Fig. 1C: e). Torsion-induced apoptotic cells were most obvious in intra-tubular elements, but a few interstitial components were also observed. Interestingly, the apoptotic germ cells were found to be located at the periphery of each tubule (Fig. 1C: b–d), which are the spermatogonia. The percentages of TUNEL-positive seminiferous tubules of the ipsilateral sham, 1, 2 and 4 h groups were 10.2 ± 1.4, 12.3 ± 0.9, 16.4 ± 1.8 and 25.3 ± 4.4%, respectively. Compared with the contralateral testis in the 2 and 4 h groups, torsion significantly induced apoptosis in the ipsilateral testis. There were no statistically significant differences among the contralateral groups.

### Torsion impaired the testosterone secretion capacity of TICs and LCs

**Figure 2:**
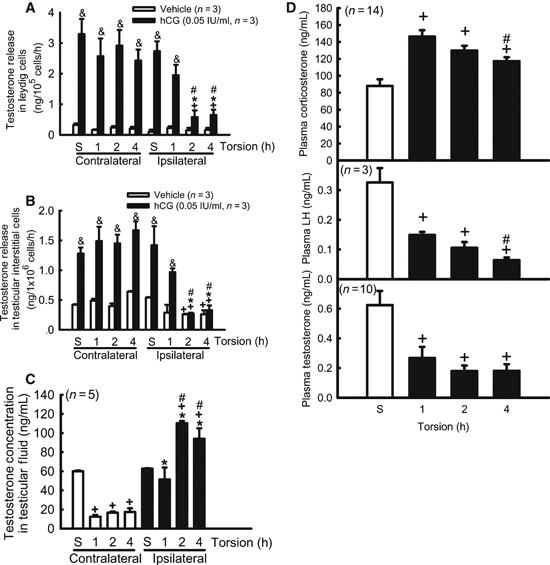
The effects of torsion on testosterone, corticosteroid and luteinizing hormone (LH) levels. Torsion impaired testosterone secretion capacity of (A) Leydig cells and (B) testicular interstitial cells. (C) The testosterone levels of testicular interstitial fluid were enhanced in the ipsilateral testes, but suppressed in the contralateral testes after unilateral torsion. (D) Experimental torsion increased plasma corticosterone levels and reduced plasma LH levels, which contributed to the decline in plasma testosterone levels. Values are mean ± SEM. *p<0.05, vs. respectively contralateral testes; ^+^*p*<0.05, vs. sham (S) group; ^#^*p*<0.05, vs. ipsilateral 1 h group; ^&^*p*<0.05, vs. counterpart vehicle treatment.

The hCG-evoked testosterone secretion of the TICs and LCs was significantly reduced after 2 and 4 h torsion. In the LCs, there was approximately 29, 79 and 76% suppression of the hCG-evoked testosterone levels noted in the 1, 2 and 4 h groups as compared with the ipsilateral sham group. The hCG-evoked testosterone levels were 27.4-, 8.9-, 3.9- and 4.1-fold greater than the respective vehicle levels of the ipsilateral testes (Fig. 2A). In the TICs, the hCG-evoked testosterone levels of the 1, 2 and 4 h groups were approximately 32, 81 and 77% lower than the ipsilateral sham group. The hCG-evoked testosterone levels were 2.6-, 3.3-, 1.0- and 1.2-fold greater than the respective vehicle levels of the ipsilateral testes (Fig. 2B). However, the basal testosterone levels of either the LCs or the TICs were not significantly different across all groups.

### Torsion altered the testosterone levels of the TIF

The TIF testosterone concentration was examined by RIA (Fig. 2C). The TIF testosterone levels of the ipsilateral testes were 1.7- and 1.5-fold increased after 2 and 4 h of torsion. The TIF testosterone levels of the contralateral testes at 1, 2 and 4 h of torsion were approximately 76, 68 and 67% lower than that in the sham group.

### Estimation of hormone levels

Torsion increased the plasma corticosterone concentration. The corticosterone plasma levels after sham, 1, 2 and 4 h of torsion were 88 ± 7.8, 146.3 ± 7.6, 129.8 ± 5.6 and 117.2 ± 4.7 ng/mL, respectively (Fig. 2D). Torsion suppressed plasma LH and testosterone levels. The plasma LH concentrations after sham, 1, 2 and 4 h of torsion were 326 ± 48, 148 ± 10, 105 ± 19 and 63 ± 9 pg/mL, respectively. The plasma testosterone levels after sham, 1, 2 and 4 h of torsion were 0.63 ± 0.10, 0.26 ± 0.08, 0.18 ± 0.02 and 0.17 ± 0.03 ng/mL, respectively (Fig. 2D).

### Torsion increased the mRNA and protein expression of SGK1

**Figure 3:**
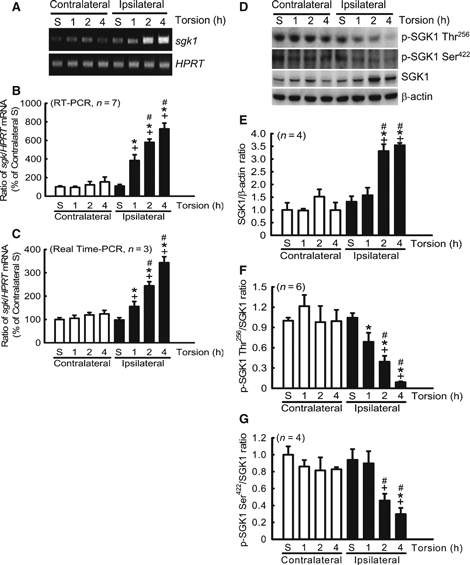
Torsion increased the expressions of serum- and glucocorticoid-inducible kinase-1 (SGK1) mRNA and protein, but suppressed SGK1 phosphorylation. Expression of *sgk1* mRNA was increased by 2 and 4 h of torsion. (A) A representative gel pattern shows the expression of *sgk1* mRNA for the bilateral testes after different periods of torsion. Quantitative comparisons of *sgk1* mRNA were carried out by (B) reverse transcription-polymerase chain reaction (PCR) and (C) real-time PCR. Hypoxanthine phosphoribosyltransferase (*HPRT*) gene was used as an internal control. The values show the ratio of *sgk1/HPRT* as mean ± SEM. (D) A representative gel pattern shows expressions of SGK1, p-SGK1 Thr^256^ and p-SGK1 Ser^422^ in the bilateral testes following torsion. SGK1 protein expression was increased in the 2- and 4-h torsion groups compared with the sham group and in the ipsilateral testis (B), but expression of (C) p-SGK1 Thr^256^and (D) p-SGK1 Ser^422^ was decreased in the ipsilateral testes after torsion. Blotting shows SGK1 at 50 kDa, p-SGK1 Thr^256^ and Ser^422^ at 48 kDa and β-actin at 43 kDa. β-actin was used as an internal control. Values are mean ± SEM. *p<0.05, vs. respectively contralateral testes; ^+^*p*<0.05, vs. sham (S) group; ^#^*p*<0.05, vs. ipsilateral 1 h group.

The expressions of *sgk1* mRNA were determined by RT-PCR and real-time PCR. With RT-PCR using agarose gel quantitative comparison, the *sgk1* mRNA expression of the 1, 2 and 4 h ipsilateral testes showed a 3.6–6.7-fold increase compared with the sham group (Fig. 3A,B). By real-tme PCR, the *sgk1* mRNA expression of the 1, 2 and 4 h ipsilateral testes showed a 1.6–3.5-fold increase compared with the sham group (Fig. 3C). Across all the groups, there were no significant differences in the amount of *sgk1* mRNA expression by the contralateral testes using either RT-PCR or real-time PCR.

A representative gel pattern showing the protein expression of SGK1 after torsion is shown in Fig. 3D. SGK1 protein expression was enhanced in the 2 and 4 h torsion groups, showing a 2.5-fold increase in the ipsilateral testes compared with the sham group (Fig. 3E). Thus, the expression of SGK1 at both the mRNA and protein levels is significantly increased after torsion.

### Torsion decreased SGK1 phosphorylation at Thr^256^ and Ser^422^

The expressions of p-SGK1 Thr^256^ and Ser^422^ were investigated by immunoblotting. In the ipsilateral testes, the p-SGK1 Thr^256^ levels of the 1, 2 and 4 h torsion groups were approximately 34, 62 and 91% lower than those in the sham group (Fig. 3F), respectively. The p-SGK1 Ser^422^ levels of the ipsilateral testes in the 1, 2 and 4 h torsion groups were approximately 5, 69 and 75% lower than those in the sham group (Fig. 3G), respectively. Taken together, it was found that torsion suppressed the phosphorylation of SGK1.

### Torsion decreased the phosphorylation of PDK1

**Figure 4:**
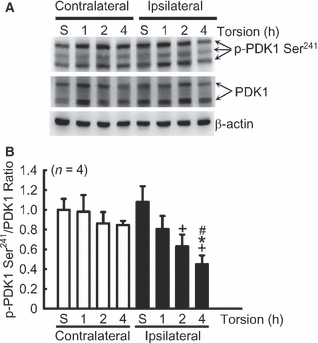
Torsion decreased the phosphorylation of phosphoinositide-dependent protein kinase-1 (PDK1). (A) A representative gel pattern shows the expression of p-PDK1 Ser^241^ and PDK1 after different periods of torsion. Blotting shows PDK1 (two bands) and p-PDK1 Ser^241^ (three bands) at 58–68 kDa, and β-actin at 43 kDa. β-actin was used as an internal control. (B) The ratio of p-PDK1 Ser^241^/PDK1 was decreased after 2 and 4 h of torsion in the ipsilateral testis. *p<0.05, vs. respectively contralateral testes; ^+^*p*<0.05, vs. sham (S) group; ^#^*p*<0.05, vs. ipsilateral 1 h group.

A representative gel pattern displaying the expressions of PDK1 and the p-PDK1 Ser^241^ is shown in Fig. 4A. The ratios of p-PDK1 Ser^241^/PDK1 in the ipsilateral testes of the 1, 2 and 4 h groups were approximately 25, 41 and 58% lower than those in sham group, respectively. The p-PDK1 Ser^241^/PDK1 ratio of the ipsilateral testis was suppressed compared with the contralateral testis after 4 h of torsion (Fig. 4B). There were no differences among any of the groups in terms of the contralateral testes. Thus, torsion significantly decreased the phosphorylation of PDK1.

### Torsion decreased the FOXO3a phosphorylation

**Figure 5:**
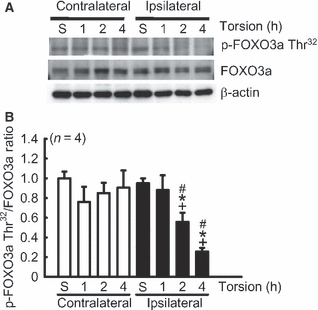
Torsion decreased the phosphorylation of FOXO3a. (A) A representative gel shows the expression of FOXO3a and p-FOXO3a Thr^32^ after torsion. Blotting shows FOXO3a at 90 kDa, p-FOXO3a Thr^32^ at 95 kDa and β-actin at 43 kDa. β-actin was used as an internal control. (B) The ratio of p-FOXO3a Thr^32^/FOXO3a in the ipsilateral testis was decreased after 2 and 4 h of torsion. ^*^p<0.05, vs. respectively contralateral testes; ^+^*p*<0.05, vs. sham (S) group; ^#^*p*<0.05, vs. ipsilateral 1 h group.

Immunoblotting was performed for p-FOXO3a Thr^32^, the preferential phosphorylation site of SGK1 (Fig. 5A). The p-FOXO3a Thr^32^/FOXO3a ratio in the ipsilateral testes of the 1, 2 and 4 h groups was approximately 7, 42 and 74% lower than that in sham group, respectively. Thus, torsion suppressed the expression of p-FOXO3a Thr^32^ in the ipsilateral testis over a 2-h period compared with the contralateral testis (Fig. 5B).

### Decrease in SGK1 and p-FOXO3a Thr^32^ expression in apoptotic cells

**Figure 6:**
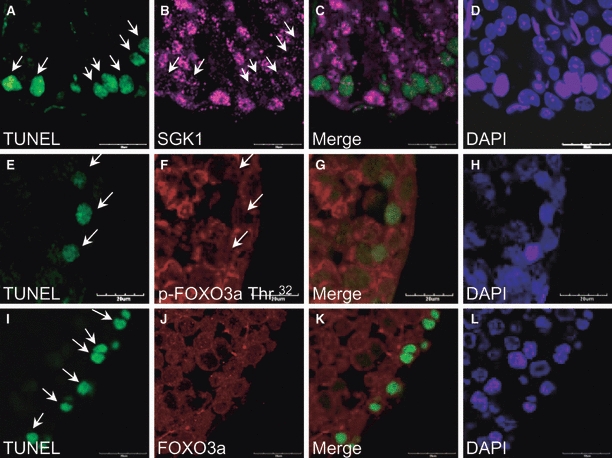
Immunofluorescent terminal deoxynucleotidyl transferase-mediated dUTP-biotin nick-end labelling (TUNEL) staining of serum- and glucocorticoid-inducible kinase-1 (SGK1)/TUNEL, p-FOXO3a Thr^32^/TUNEL and FOXO3a/TUNEL in the ipsilateral 4-h testis sections. The apoptosis cells (arrows) are pinpointed by TUNEL stain (green; A, E, and I), whereas the expression of SGK1 (purple; B) and p-FOXO3a Thr^32^ (red; F) is barely detectable. The expression of FOXO3a (red; J) is not directly relatable to identifiable apoptotic cells. The colocalization images of SGK1/TUNEL, p-FOXO3a Thr^32^/TUNEL and FOXO3a/TUNEL are shown in C, G and K. The sections are counterstained with DAPI (blue; D, H and I). Scale bar: 20 μm.

The colocalization of SGK1/TUNEL, p-FOXO3a Thr^32^/TUNEL and FOXO3a/TUNEL was detected by immunofluorescence microscopy in paraffin testicular sections (Fig. 6). Torsion-induced apoptotic cells can be identified by TUNEL stain in 4-h ipsilateral testis (Fig. 6A,E,I), whereas the expression of SGK1and p-FOXO3a Thr^32^ was barely detectable in the apoptotic cells (Fig. 6B,F). In addition, expression of FOXO3a (Fig. 6J) cannot be directly correlated with cells undergoing apoptosis.

## Discussion

The results from this in vivo study support our hypothesis that testicular ischaemia-induced apoptosis is associated with the PDK1, SGK1 and FOXO3a signalling cascade. Our results suggest, after torsion stimulation, that the PDK1/SGK1/FOXO3a signalling cascade was downregulated, that the plasma corticosteroid level was increased, that plasma LH and testosterone levels were decreased and that the testosterone-secreting capacity of LCs and TICs was impaired.

The in vivo testicular ischaemic model in this study successfully led to testicular oedema, haemorrhage, congestion and cyanosis in the ipsilateral testis. Moreover, we noted that the MSTD was significantly decreased after 2 h of torsion, which is known to be an early sensitive indicator (Karaguzel *et al.*, 1995; Zhang *et al.*, 2002) of the testicular size, which has a positive correlation with testicular function in torsion (Takihara *et al.*, 1987). It is well known that venous obstruction leads to hypoxia, poor nutrient supply and metabolic product accumulation in local testicular elements; the capillary permeability and hydrostatic pressure may be changed in testes (Altay *et al.*, 2001). Therefore, torsion-induced interstitial oedema might alter the MSTD.

It is well known that the testicular torsion-induced damage severity depends on the duration and degree of the spermatic cord twisting. A previous study indicated that necrosis was only found in one rat immediately after 4 h of 1080° torsion (Romero *et al.*, 2009). This group of rats underwent more severe torsion than the rats in our study group. Although we did not evaluate the extent of necrosis in the present experimental setting, it is reasonable to speculate that the extent of torsion-induced necrosis may well be less with 720° torsion than with 1080° torsion. Therefore, it is likely that the level of necrosis in our study will be small, if detectable at all.

In addition, torsion induced a progressive loss of testicular components, depending on cell type and ischaemic duration. We found that the TUNEL-positive staining mainly occurred in spermatogonia, although a few interstitial cells were also observed to be apoptotic after 2 h of torsion. This result is consistent with that of the previous reports in which LCs were less sensitive to ischaemic stimuli than spermatogenic cells (Anderson & Williamson, 1990; Arena *et al.*, 2006). Turner *et al.* (2005) reported that torsion following by detorsion resulted in no LC apoptosis and that the twisted testis maintained intra-testicular testosterone levels with considerable steroidogenic capacity (Baker & Turner, 1995). However, our results revealed that hCG-induced testosterone secretion, one measure of the functional capacity of testosterone production, was significantly reduced in the LCs and TICs of the ipsilateral testes immediately after 2 h of torsion without detorsion, but that no effect on the basal testosterone secretion of LCs and TICs was detected between the bilateral testes. Although blood flow to the testis is occluded completely by the 720° spermatic cord twisting during the time of torsion (Turner, 1985), testosterone production was accompanied by basal testosterone release, which results in increased levels of TIF testosterone during mechanical stimuli in the ipsilateral testis following torsion.

In addition, an increase in plasma corticosterone level and a dramatic decrease in LH concentration were observed after torsion. This LH reduction might be caused by the stress-suppressed hypothalamus–pituitary–gonadal axis via increased Rfamide-related peptides and corticosterone production (Rivier & Rivest, 1991; Kirby *et al.*, 2009) that inhibit secretion of gonadotropin-releasing hormone. It is reasonable to speculate that the reduction in LH may contribute to the decline to 50% of the sham group's contralateral TIF testosterone and plasma testosterone concentrations after 1 h of torsion.

Previous studied have revealed that SGK1 expression is altered by hypoxia/ischaemia stimulations. Aoyama *et al.* (2005) report that expressions of SGK1 and p-SGK1 Thr^256^ are elevated under hypoxia treatment, but lowered by serum deprivation in cardiomyocytes in vitro*.* We therefore speculated that SGK1 expression is altered by testicular ischaemia in vivo. However, previous reports have indicated that *sgk1* gene expression in testicular tissue is very low and barely detectable when examined by Northern blotting (Webster *et al.*, 1993; Waldegger *et al.*, 1998). Using RT-PCR and real-time PCR, we found that *sgk1* mRNA was abundantly present in rat testes. We also noted that the levels of SGK1 mRNA and protein were significantly increased after torsion. A previous study has revealed that androgen treatment increased SGK1 protein expression (Shanmugam *et al.*, 2007). In this in vivo study, the expression of SGK1 in the ipsilateral testis is induced by torsion. This induction might relate to the increase in TIF testosterone levels after torsion, which has been described before. Our results provide new evidence for ischaemia-induced damage in vivo. In addition, previous studies have indicated that SGK1 expression can also be induced by ischaemia/reperfusion in vivo. Expression of the *sgk1* gene is induced by ischaemia/reperfusion in brain (Lu *et al.*, 2003; Nishida *et al.*, 2004). Moreover, Rusai *et al.* (2009) indicated that SGK1 is upregulated after ischaemia and reaches its plateau after 6 h of reperfusion in kidney. In addition, it has been found that both phospho-SGK1 and total SGK1 increased 2–7 days after ischaemia/reperfusion in the cardiomyocytes (Aoyama *et al.*, 2005)*.*

In contrast, our results show that levels of p-SGK1 Thr^256^ and p-SGK1 Ser^422^ were suppressed after testicular ischaemia. Related to these differences in SGK1 and p-SGK1 levels after testicular ischaemia, it was noted that the expression of p-PDK1 Ser^241^ was decreased after 2 h of torsion. PDK1 is the kinase mainly responsible for SGK1 activation (Kobayashi & Cohen, 1999). Budas *et al.* (2006) found that inhibition of the ability to phosphorylate PDK1 results in a larger heart infarcted area after ischaemia/reperfusion. The mediation of cell survival by Ser^241^ phosphorylation needs the activation of PDK1 (Casamayor *et al.*, 1999), and is altered under ischaemic stress affecting the kidney (Zheng *et al.*, 2008) and the myocardium (Kis *et al.*, 2003); this occurs via the regulation of phosphoinositide-3-kinase (PI3K) activity. Moreover, a previous report has revealed that testosterone suppresses PI3K activity (Papadopoulou *et al.*, 2008), which results in a decrease in PDK1 phosphorylation. We therefore speculate that torsion-induced apoptosis may work together with PDK1 and SGK1 dephosphorylation in rats via increasing levels of TIF testosterone.

FOXO3a, a pro-apoptotic factor, is negatively regulated by SGK1. It has been shown that SGK1 phosphorylates the Thr^32^ of FOXO3a selectively (Brunet *et al.*, 2001). When mutated at the SGK1 phosphorylation site, activation of FOXO3a leads to apoptosis, which is mediated by a decrease in SGK1 phosphorylation (Brunet *et al.*, 2001). A recent report has shown that decreased phosphorylation of FOXO3a induces transcription of genes that promote cell-cycle arrest (p21 and p27) and apoptosis (Bim, FasL and TRAIL); this occurs by inhibiting ubiquitination, which inhibits degradation by the 26S proteasome (Yang *et al.*, 2008). In this in vivo study, torsion increased SGK1 at both the mRNA and protein levels, but p-SGK1 Thr^256^ and p-SGK1 Ser^422^ were simultaneously reduced. This phenomenon may amplify the effects of SGK1 dephosphorylation and lead to FOXO3a dephosphorylation, then apoptosis. The expression of p-FOXO3a Thr^32^ was significantly decreased by 2 and 4 h of torsion; this is similar to the findings of a previous report, where chemical anoxic exposure inhibited p-FOXO3a Thr^32^ in LLC-PK_1_ cells (renal epithelial cell line from porcine kidneys) (Andreucci *et al.*, 2003) in vitro. Moreover, using immunofluorescence microscopy, we found that apoptotic spermatogonia show a directly related decrease in expression of SGK1 and p-FOXO3a Thr^32^, but that there is no significant increase in the expression of FOXO3a. This decrease in p-FOXO3a Thr^32^ would seem to be related to the dephosphorylation of PDK1 and SGK1, clearly highlighting the importance of decreased phosphorylation of FOXO3a during torsion.

**Figure 7:**
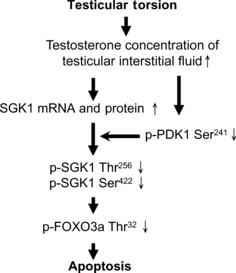
Schematic signalling model of the phosphoinositide-dependent protein kinase-1 (PDK1)/serum- and glucocorticoid-inducible kinase-1 (SGK1)/FOXO3a pathway during testicular torsion. We propose that testicular torsion decreases SGK1 phosphorylation via a decrease in PDK1 phosphorylation, which then appears to downregulate FOXO3a phosphorylation and this leads to cell apoptosis.

The PDK1, SGK1 and FOXO3a signalling cascade seems to be affected by various stimuli (Kobayashi & Cohen, 1999; Brunet *et al.*, 2001; Leong *et al.*, 2003), including ischaemic stimulation following apoptosis. Necrosis is no longer considered the sole mechanism for ischaemia-induced cell death, and it seems likely that apoptosis is an important mode of cell death, en route to ultimate cell death, in this situation. We therefore suggest that testicular ischaemia downregulates the phosphorylation of the PDK1/SGK1/FKHRL1 signalling pathway and this induces cell apoptosis, as shown in Fig. 7.

In a clinical context, the optimal time for torsion/detorsion surgery has been suggested to be 4–6 h after torsion has incurred. However, the results of this animal study suggest that torsion-induced spermatogonia apoptosis occurs at least partially via the PDK1/SGK1/FOXO3a signalling cascade and impairs the testosterone secretion capacity of the testes after only 2 h of torsion. In this context, we suggest that medical treatment, such as drug administration and reperfusion surgery, should be performed as early as 2 h after testicular ischaemia has occurred to prevent greater damage.
